# SNHG15 Positively Regulates Influenza Virus Infection Through Its Association With miR‐153 and RABL2A

**DOI:** 10.1002/jmv.71084

**Published:** 2026-07-23

**Authors:** Samuel Pushparaj, Kishore Vaddadi, Chaoqun Huang, Yurong Liang, Dao Xu, Gayan Bamunuarachchi, Prince Jhandai, Keerthana Santhosh, Elizabeth Varghese, Lin Liu

**Affiliations:** ^1^ Oklahoma Center for Respiratory and Infectious Diseases Oklahoma State University Stillwater Oklahoma USA; ^2^ The Lundberg‐Kienlen Lung Diseases and Infection Laboratory, Department of Physiological Sciences Oklahoma State University Stillwater Oklahoma USA

**Keywords:** host factors, influenza virus, long non‐coding RNAs, miR‐153, proviral, RABL2A, SNHG15

## Abstract

Influenza viruses cause seasonal epidemics, and current vaccines do not fully protect against them. This study focuses on long non‐coding RNAs (lncRNAs), which are RNA molecules that do not code for proteins but play crucial roles in various biological processes. We identified 17 lncRNAs altered by the influenza virus. Among these, SNHG15 was found to enhance influenza virus infection. Knockdown of SNHG15 using CRISPR interference and RNA interference reduced influenza virus infection, while overexpression of SNHG15 increased virus replication. We found that miR‐153‐3p, a competitive endogenous RNA (ceRNA) for SNHG15, acts as an anti‐influenza microRNA, and its overexpression negated SNHG15's proviral activity. RNA pull‐down and mass spectrometry identified Rab‐like protein 2A (RABL2A) as an interacting partner of SNHG15. Both SNHG15 and RABL2A facilitated influenza virus internalization. Additionally, siRNA‐mediated knockdown of RABL2A reduced SNHG15‐mediated virus internalization. In conclusion, SNHG15 enhances influenza A virus infection through its interactions with miR‐153 and RABL2A.

## 1 Introduction

Influenza virus is an enveloped RNA virus that belongs to the orthomyxoviridae family that has a negative single‐stranded segmented genome [1]. Four influenza virus serotypes, A–D, have been identified so far [2]. Influenza A virus (IAV) and Influenza B virus are the major types that affect the human population causing seasonal epidemics and occasional pandemics [3]. Influenza C virus causes mild infection in humans and is endemic whereas influenza D virus primarily affects cattle and is not known to infect humans [4].

IAV genome comprises of eight viral nucleoprotein (vRNP) segments that give rise to 11+ viral proteins [5, 6]. The viral RNA is wound on NP viral proteins. Hemagglutinin (HA) is responsible for the attachment of the virus to the sialic acid receptor present on the cell surface. Upon its binding, viral particles are internalized into the cell through receptor‐mediated endocytosis. This process can also be through clathrin‐mediated, caveolae‐mediated, or clathrin‐ and caveola‐independent endocytosis and micropinocytosis [7, 8]. Several host factors have been found to aid the internalization of influenza virus particles. Mutation in the N‐acetylglucosaminyltransferase I gene results in the failure of influenza virus internalization, indicating that besides sialic acid attachment, the N‐linked glycoprotein is required for the successful entry of influenza virus into cell [9]. Upon virus attachment, lipid raft clustering is formed, which in turn activates the epidermal growth factor receptor and downstream signaling molecules such as phosphatidylinositol 3‐kinase and phospholipase C γ1, and ultimately leads to the internalization of influenza virus particles [10, 11, 12]. Epsin 1 is a cargo‐specific adaptor that facilitates the clathrin‐mediated entry of the influenza virus [13]. The G Protein‐Coupled Receptor, Free Fatty Acid Receptor 2 (FFAR2), enhances influenza virus clathrin‐mediated endocytosis through its interaction with β‐arrestin1 and Adaptor Related Protein Complex 2 Subunit Beta 1 (AP2B1) [14]. Matrix protein 2 (M2) through its selective proton ion channel activity enables the release of viral ribonucleoproteins (vRNPs) into the cytoplasm [15]. The replication of the influenza viral particle takes place in the nucleus. The polymerase complex includes Polymerase acidic protein (PA), Polymerase basic protein 1 (PB1), and Polymerase basic protein 2 (PB2). Neuraminidase (NA) cleaves the sialic acid receptor, and the progeny particles egress out of the cell. 18 HA and 11 NA subtypes have been identified so far in nature [16].

IAV replication is regulated through a variety of host factors, including non‐coding RNAs [17, 18]. Long non‐coding RNAs (lncRNAs) are RNA transcripts with > 200 nucleotides without protein‐coding potential [19]. Several lncRNAs have been identified to regulate influenza virus replication in vitro and/or in vivo models. However, only a few conserved lncRNAs have been identified [20]. LncRNAs exert functional activity by various mechanisms through binding with DNA, RNA, and protein [19]. Most lncRNAs have been found to regulate host antiviral immune response by transcriptional regulation. LncRNAs, MxA, and TSPOAP1‐AS1 negatively regulate IFNβ and interferon‐stimulated genes (ISG) expression through DNA binding, resulting in the enhancement of IAV replication [21, 22]. LncRNA ISG20 suppresses IAV replication by binding miRNA‐326 and positively regulating ISG 20 expression [23]. LncRNA AVAN acts as a scaffold and facilitates the interaction of TRIM 25 and RIG‐I, resulting in the ubiquitination of RIG‐I and inhibition of viral replication [24]. LncRNA PAAN interacts with PA viral protein and promotes the assembly of viral RNA polymerase [25]. LncRNA IPAN interacts and stabilizes PB1 viral protein, resulting in enhanced viral replication [26]. LncRNA IVRPIE also boosts the transcription of interferon β1 and several ISGs by modifying their histones and limits IAV replication [27].

LncRNA IFITM4P serves as a positive regulator of innate antiviral immunity. Its ectopic expression suppresses IAV replication, while deficiency enhances viral production [28]. Knockdown of LINC02574 enhances IAV infection by decreasing type I and III IFNs, ISGs, and STAT1 activation, and impairs RIG‐I, TLR3, and MDA5 expression, reducing IRF3 phosphorylation [29]. Lnc‐PINK1‐2:5, a nuclear lncRNA, upregulates TXNIP, which reduces influenza infection. Knockdown of TXNIP negates Lnc‐PINK1‐2:5's antiviral effects [30].

LncRNAs may not exhibit the same pattern of conservation as seen in protein‐coding genes. The presence of conserved secondary structures may indicate functional significance, as sequence conservation is less common in lncRNAs [31, 32]. Only a handful of conserved lncRNAs namely MicroRNA 155 Host Gene (MIR155HG), Interferon‐Stimulated Long Noncoding RNA (LncRNA ISR), Nuclear Enriched Abundant Transcript 1 (NEAT1), and RIG‐I‐dependent IAV‐upregulated noncoding RNA (RDUR) that regulate influenza virus replication have been identified. MIR155HG is a nuclear lncRNA that is upregulated during influenza virus infection by RIG‐I and TLR3 dependent type I IFN signaling [33]. Overexpression of MIR155HG inhibits PTP1B expression, which results in a decreased IAV replication through an increased type I IFN response. As the name suggests, LncRNA ISR is a lncRNA induced by IFNβ and influenza virus infection [34]. Its expression is regulated through RIG‐I mediated NF‐κB signaling pathway. LncRNA ISR is found to be antiviral lncRNA, but its molecular mechanism of IAV regulation remains unknown. NEAT1 is considered to be a functionally conserved lncRNA but lacks sequence conservation. NEAT1 is a nuclear lncRNA that is upregulated through the TLR3 receptor upon influenza virus infection or poly (I:C) treatment [35]. During the physiological condition, NEAT1 is bound to SFPQ, a paraspeckle protein that represses the transcription of IL8. During influenza infection, NEAT1 is found to dissociate from SFPQ and binds to the promoter region of IL8, resulting in the activation of IL8 transcription. RDUR is a newly identified conserved lncRNA that was upregulated both in vitro and in vivo [36]. Its expression is mediated through the RIG‐I‐dependent NF‐κB signaling pathway, similar to LncRNA ISR. RDUR exhibits antiviral activity on influenza virus through positively regulating IRF3 activation and upregulating ILF2/ILF3 resulting in increased IFN response. RDUR knockout mice were found to be more susceptible to IAV infection. Mice lacking RDUR show the activation of NF‐κB and enhanced inflammatory response after influenza virus infection.

In this study, we aimed to identify differentially expressed lncRNAs that are conserved in humans and mice and to determine their regulatory role on influenza virus infection. We identified a conserved lncRNA, Small Nucleolar RNA Host Gene 15 (SNHG15) that was upregulated in a dose and time‐dependent manner during influenza virus infection. Characterization studies found that SNHG15 was found predominantly in the cytoplasm and lacks the protein‐coding potential. Functional studies revealed that SNHG15 is a potent proviral lncRNA that competes with an anti‐influenza miR‐153‐3p and enhances influenza virus internalization into the cells through its association with RABL2A protein.

## 2 Results

### 2.1 Identification of Conserved lncRNAs Dysregulated During Influenza Virus Infection

We have previously performed RNA sequencing analysis of human lung epithelial A549 cells infected with influenza A/Purerto Rico/8/34 (PR8) virus and identified 1914 differentially expressed lncRNAs [37]. To identify sequence‐conserved (orthologous) lncRNAs dysregulated during IAV infection, we chose a total of 209 upregulated and 229 downregulated lncRNAs in human cells with a fold change of ≥ 5 and assessed for their conservation in mice using LncRNAtor database and Mouse Dec. 2011 (GRCm38/mm10) Assembly [38]. The conservation referred to in this study is sequence conservation of orthologous human and mouse lncRNA loci, rather than structural or functional conservation. We found that 6 upregulated and 11 downregulated lncRNAs are conserved between humans and mice (Supplementary Table 1). Utilizing publicly available RNA sequencing datasets [39], we then determined whether these conserved lncRNAs were altered in the lungs of two strains of mice, C57BL/6 J and AJ mice that were infected with PR8 at 2 and 4 days post‐infection (dpi). Among the 17 lncRNAs, only three lncRNAs, namely MIR155 host gene (MIR155HG), MIR22 host gene (MIR22HG), and small nucleolar RNA host gene 15 (SNHG15) were upregulated in mice by influenza virus infection (Supplementary Table 2).

**Figure 1:**
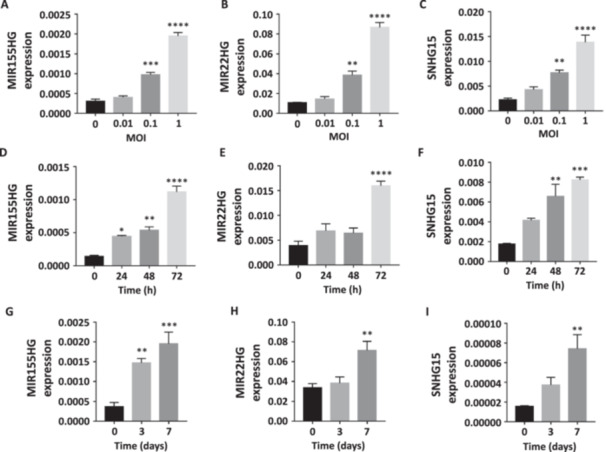
Validation of dysregulated conserved lncRNAs during influenza infection. A549 cells were infected with mock or PR8 at indicated doses for 24 h (A–C), or at an MOI of 0.01 for indicated hours post infection (hpi) (D–F). C57BL/6 J mice were infected with 250 PFU of PR8 for 3‐ and 7‐days post infection (dpi) (G–I). MIR155HG, MIR22HG and SNHG15 expression levels in PR8‐infected A549 cells and lung tissues were determined by real‐time PCR. Data were normalized to β‐actin and were expressed as means ± SE. *p < 0.05, **p < 0.01, ***p < 0.001, ****p < 0.0001 vs 0 MOI or 0 time (*n* = 3 for cells and *n* = 4 for animals). One‐way ANOVA, followed by Tukey's comparison.

To further validate the dysregulation of the selected lncRNAs, we infected A549 cells with PR8 virus at MOI 0, 0.1, 0.01, and 1 for 24 h and then determined the lncRNA levels by real‐time PCR. MIR155HG, MIR22HG, and SNHG15 levels were found to be significantly upregulated at MOI 0.1 and 1 (Figure 1A–C). We then examined the kinetics of lncRNA dysregulation in A549 cells by infecting them with PR8 at a low MOI of 0.01 for 24, 48, and 72 h. We observed that MIR155HG and SNHG15 exhibited a time‐dependent upregulation during PR8 virus infection (Figure 1D,F). MIR22HG induction was observed only at 72 h post‐infection (hpi), but not at 24 and 48 hpi (Figure 1E). Since all three lncRNAs are conserved in mice, we further confirmed their dysregulation during influenza virus infection in C57BL/6 J mice infected with a sub‐lethal dose of PR8 virus for 0, 3, and 7 dpi. MIR155HG levels were upregulated at 3 and 7 dpi (Figure 1G). A significant induction of SNHG15 and MIR22HG was observed at 7 dpi (Figure 1H,I).

MIR155HG and MIR22HG host miR‐155 and miR‐22 sequences within its exon and are upregulated during influenza infection [33, 40]. MIR155HG suppresses influenza virus replication through inhibition of protein tyrosine phosphatase 1B (PTP1B) and increased production of interferon‐beta (IFN‐β) [33]. Both MIR155HG and MIR22HG encode endogenous micropeptides [40, 41]. SNHG15 was first identified as a short‐lived lncRNA that was stabilized upon chemical stress [42]. SNHG15 is a critical regulator of tumor progression in several human cancers and is viewed as a potential biomarker for cancers [43]. SNHG15 is also upregulated during retinal infection with *Toxoplasma gondii* in humans [44]. The role of SNHG15 in influenza virus infection has not been studied yet. Thus, we chose SNHG15 for further studies.

### 2.2 Characterization of SNHG15

**Figure 2:**
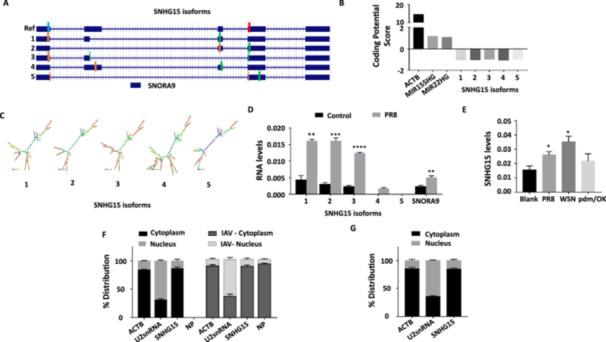
Characterization and regulation of SNHG15 during influenza infection. (A) According to the Human Dec. 2013 Assembly (GRCh38/hg38), SNHG15 has five annotated isoforms and encodes SNORA9 in the intron region (A). Common forward primer (blue) and reverse primer (red) were indicated in the reference (ref) sequence. Isoform specific forward primers (orange) and reverse primers (green) were indicated in the respective isoforms. (B) Coding potential scores for mRNA, and lncRNAs were calculated using the Coding Potential Calculator (http://cpc.gao-lab.org/). (C) RNA structures of SNHG15 isoforms were predicted by the RNAfold web server. (D) A549 cells were infected with mock or PR8 at an MOI of 1 for 24 h. (E) HEK 293 cells were infected with indicated strains at a MOI of 0.1 for 24 h. SNHG15 expression levels were determined by real‐time PCR and normalized to β‐actin. (F, G) Subcellular localization of SNHG15 in A549 cells with or without PR8 infection at an MOI of 1 for 24 hpi (F) and in HBTE cells without virus infection (G). Data were expressed as means ± SE. *p < 0.05, **p < 0.01, ***p < 0.001, ****p < 0.0001 versus control or blank (*n* = 3). Student's *t*‐test for D and One‐way ANOVA, followed by Tukey's comparison for E.

SNHG15 encodes small nucleolar RNA 9 (SNORA9) in its intron between exons 2 and 3 of the reference gene (Figure 2A). According to the human genome, GRCh38/hg38 assembly, SNHG15 has five known isoforms. SNHG15 isoform 1 (NR_003697.2) and isoform 4 (NR_152596.1) have five exons with 983 base pairs (bp) and 860 bp, respectively. Isoform 2 (NR_152594.1) and isoform 3 (NR_152595.1) have four exons with a similar size (783 and 774 bp). Isoform 5 (NR_152597.1) is the smallest isoform (713 bp) with only three exons. Unlike MIR155HG and MIR22HG that have the ability to code for micropeptides, all SNHG15 isoforms lack coding potential (Figure 2B) as predicted using Coding Potential Calculator (http://cpc.gao-lab.org/).

The secondary structures of SNHG15 isoforms were predicted using the RNAfold web server (http://rna.tbi.univie.ac.at/cgi-bin/RNAWebSuite/RNAfold.cgi). They share highly similar secondary loop structures since all the isoforms share the same sequence in exon 1, 4, and 5 in reference to isoform 1 (Figure 2C).

In the results above (Figure 1), we used a common primer pair (blue and red) as indicated in the reference gene in Figure 2A to detect all the SNHG15 isoforms. This primer pair was used to determine SNHG15 levels thereafter unless noted otherwise. To distinguish the expression levels of each SNHG15 isoform, we designed isoform‐specific primer pairs (Figure 2A) and measured their expression levels in lung epithelial A549 cells using real‐time PCR. Though the location of a few primers seems to overlap, their sequences were unique to the isoforms in the primer locations. SNHG15 isoforms 1, 2, and 3 had higher basal expression levels compared to isoform 4, whereas isoform 5 showed negligible expression (Figure 2D). Except for isoform 5, all isoforms were upregulated by PR8 at an MOI of 1 for 24 h. SNORA9 mRNA levels were also increased by PR8 infection. The upregulation of SNHG15 was also observed in HEK 293 cells infected with various strains of IAV, including PR8 (IAV/Puerto Rico/8/1934 H1N1), WSN (IAV/WSN/1933 H1N1), and pdm OK (A/Oklahoma/3052/2009) at MOI 0.1 for 24 h, although pdm Ok did not reach a significant level (Figure 2E), suggesting that the upregulation is not strain‐specific.

To understand the functional role of SNHG15, it is important to know its subcellular localization in the cells with or without IAV infection. SNHG15 levels in cytoplasmic and nuclear fractions were quantitated by real‐time PCR. ACTB mRNA and U2snRNA were used as the positive controls for cytoplasmic and nuclear fractions, respectively. 86 ± 0.8% of SNHG15 was present in the cytoplasm of lung epithelial A549 cells, similar to that of ACTB (Figure 2F), indicating that SNHG15 is primarily located in the cytoplasm. 88 ± 0.7% of SNHG15 was also located in the cytoplasm in A549 cells infected with PR8 24 hpi (Figure 2F), indicating that SNHG15 does not translocate into the nucleus during virus infection. Since A549 cells were an immortalized cell line, we also determined the subcellular localization of SNHG15 in primary human bronchial/tracheal epithelial (HBTE) cells and found that 85 ± 0.7% of SNHG15 was present in the cytoplasm (Figure 2G).

### 2.3 Knockdown of SNHG15 Inhibits Influenza Virus Replication

**Figure 3:**
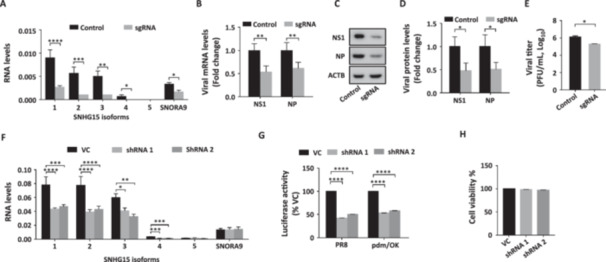
SNHG15 knockdown limits IAV replication. Stable A549‐dCAS9‐KRAB cells were transduced with an MOI of 100 of lentivirus carrying sgRNA targeting SNHG15, or its antisense control for 48 h (A). Stable A549‐dCAS9‐KRAB cells were transduced with an MOI of 100 of lentivirus carrying sgRNA targeting SNHG15, or its antisense control for 48 h and then infected with PR8 at an MOI of 0.01 for 48 h (B–E). SNHG15 isoforms and SNORA9 expression levels (A) and viral NS1 and NP RNA levels (B) were measured by real‐time PCR and normalized to β‐actin. Viral RNA levels were expressed as a ratio of the control. NS1 and NP viral protein levels were determined by Western blot, quantified and expressed a ratio of control (C, D). Viral titers in media were determined by plaque assay (E). HEK 293 cells were transfected with 1.5 µg of SNHG15 shRNAs or control vector using lipofectamine 3000 for 48 h. SNHG15 isoforms and SNORA9 expression levels were measured by real‐time PCR and normailized to β‐actin (F). HEK 293 cells were transfected with 20 ng of influenza virus luciferase reporter vector, 100 ng SNHG15 shRNAs or control vector, and 5 ng of pRL‐TK, for 24 h and then infected with PR8 at an MOI of 0.1 and pdm OK at an MOI of 0.5 for 24 h (*n* = 4). Dual luciferase activities were measured. Firefly luciferase activity was normalized to pRL‐TK *Renilla* luciferase activity. The results are expressed as a percentage of VC‐transfected cells (G). Cell viability was determined by cell titer blue assay (H). Data was shown as means ± SE. *n* = 3 independent experiments. Data was expressed as means ± SE. *p < 0.05, **p < 0.01, ***p < 0.001, ****p < 0.0001 versus control or vector control (VC) (*n* = 3). Student's *t*‐test for A, B, D, and E. One‐way ANOVA, followed by Tukey's comparison for F, G, and H.

To determine the functional activity of SNHG15 on influenza virus replication, we employed the CRISPR interference (CRISPRi) approach to knockdown SNHG15. CRISPRi utilizes deactivated Cas9 (dCas9) that is fused with a transcriptional repressor. dCas9 is catalytically inactive but maintains its DNA binding ability. We designed a sgRNA that specifically targets the promoter region of SNHG15. We transduced A549 cells stably expressing dCas9‐KRAB with a lentiviral sgRNA or its control and selected with puromycin. Real‐time PCR analyses revealed an efficient knockdown in the expression of all isoforms, including SNORA9 (Figure 3A). We then infected the control and SNHG15‐knockdown cells with PR8. The knockdown of SNHG15 resulted in a decrease in viral mRNA levels of NS1 by 46.4 ± 0.1% and NP by 38.7 ± 0.1% as well as viral protein levels of NS1 by 52.4 ± 0.2% and NP by 49.2 ± 0.2% (Figure 3B–D). The viral titers in culture media were also decreased by approximately 1 log in the knockdown cells as determined by plaque assay (Figure 3E).

In addition to SNHG15, CRISPRi also knocked down SNORA9 expression. To rule out the contribution of SNORA9 to the observed effects of SNHG15 CRISPRi knockdown on IAV infection, we designed two shRNAs that target exon 4 of SNHG15 in reference to isoform 1. We transfected shRNAs individually in HEK293 cells using Lipofectamine 3000. Both shRNAs significantly knocked down SNHG15 isoforms 1‐ 4 and had no effects on SNORA9 expression (Figure 3F). We then determined the effects of shRNAs on IAV infection using an IAV luciferase reporter assay in which influenza virus replication at the transcriptional level in the cells can be measured via a reporter vector that encodes a firefly luciferase flanked with the 5′‐ and 3′‐untranslated regions of WSN NP. HEK293 cells transfected with an shRNA and the reporter vector were infected with PR8 and pdm OK at an MOI of 0.1 and 0.5, respectively for 48 h. shRNA 1 and 2‐mediated knockdown of SNHG15 decreased PR8 virus replication by 47.7 ± 0.02% and 48.6 ± 0.03%, and pdm OK replication by 46.3 ± 0.1% and 43.4 ± 0.1%, respectively (Figure 3G). To rule out that the reduction in virus replication is due to a decreased cell viability, we performed cell titer blue assay and observed that shRNAs did not affect the viability of the transfected cells (Figure 3H). These results suggest that SNORA9 does not contribute to the effects of SNHG15 knockdown on IAV infection.

### 2.4 SNHG15 Promotes Influenza Virus Replication

**Figure 4:**
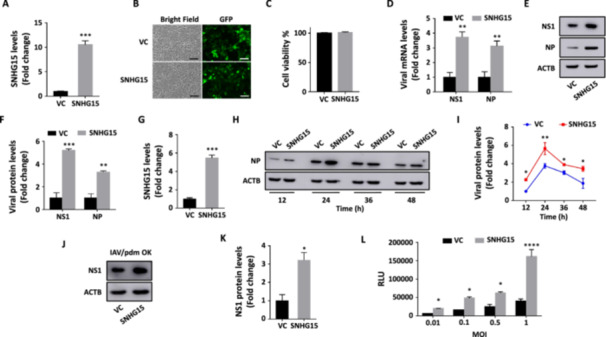
SNHG15 favors IAV replication. A549 cells were transduced with VC or SNHG15 lentivirus at an MOI of 200 (A, B), followed by infection with PR8 at an MOI of 0.01 for 48 h (D–F). A total of six fields for each condition were observed and representative Bright Field and GFP images (10 X magnification) were shown, scale bar: 100 µm (B). Stable vector control (VC) and SNHG15 cells (G–L) were infected with PR8 at an MOI of 0.01 for indicated times (H, I), 2009 pandemic H1N1 OK/09 at an MOI of 0.01 for 48 h (J, K), or PR8‐Gluc at indicated MOIs for 24 h (L). Cell viability was determined by cell titer blue assay (C). SNHG15 and viral mRNA expression levels were determined by real‐time PCR, and viral proteins were determined by Western blot and quantified. RNA and protein levels were normalized to β‐actin. Viral particle levels in cell lysates were determined by luciferase assay and expressed as relative light units (RLU) (L). Data was expressed as means ± SE. *p < 0.05, **p < 0.01, ***p < 0.001, ****p < 0.0001 versus VC (*n* = 3). Student's *t*‐test for A, D, F, G, and K. Two‐way ANOVA, followed by Tukey's comparison for I, and L.

To further confirm the proviral activity of SNHG15, we overexpressed SNHG15 in A549 cells using a lentivirus expressing SNHG15 and then infected with PR8 at an MOI of 0.01 for 48 h. Viral mRNA and proteins, as well as titers were determined. We used isoform 1 for overexpression as its basal expression is relatively higher than other isoforms. Approximately a 10‐fold increase in SNHG15 expression was achieved in the SNHG15‐overexpressed cells compared to vector control (VC) (Figure 4A). Gross microscopic examination revealed that there was not any apparent loss in cell viability, and GFP images indicated the high transduction efficiency (Figure 4B). Cell titer blue viability assay also showed that SNHG15 overexpression did not affect the cell viability (Figure 4C). SNHG15‐overexpressed A549 cells showed increased mRNA levels of NS1 and NP by 3.72 ± 0.35 and 3.13 ± 0.33 fold and increased protein levels of NS1 and NP by 5.18 ± 0.49 and 3.28 ± 0.37 fold, respectively (Figure 4D–F).

We further generated stable VC‐ and SNHG15‐overexpressed A549 cells using puromycin selection for easy downstream applications and for reducing the variations in transduction efficiency between the experiments. We infected stable VC‐ and SNHG15‐overexpressed cells with a clinical isolate, pdm OK at an MOI 0.01 for 48 h. Approximately a fivefold increase in SNHG15 expression was observed in SNHG15‐overexpressed cells compared to VC cells (Figure 4G). Similar to the results of SNHG15 overexpression with lentivirus transduction, SNHG15 stable cells increased PR8 viral NP protein levels in a time‐dependent manner (Figure 4H,I). SNHG15 also promoted pdm OK virus infection as evidenced by increases in viral NS1 mRNA and protein levels (Figure 4J,K). Using PR8 Gluc reporter virus, we observed an increase in influenza viral replication in SNHG15 stable cells at all doses of PR8 viruses (Figure 4L).

**Figure 5:**
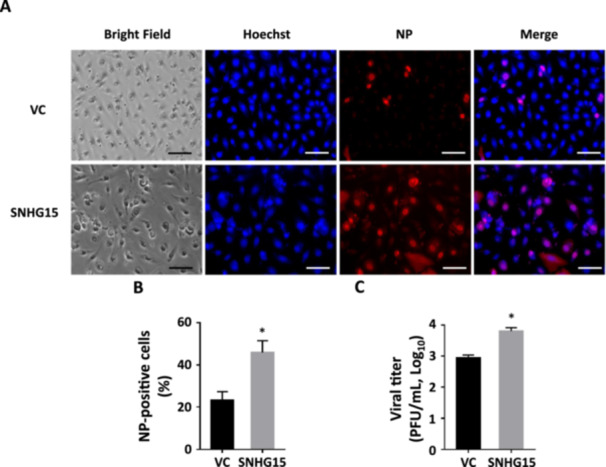
SNHG15 promotes IAV replication in primary cells. Human Bronchial Tracheal Epithelial (HBTE) cells were transduced with SNHG15 or its vector control (VC) lentivirus at an MOI of 200 for 48 h and then infected with PR8/at an MOI of 1 for 24 h. (A) NP immunofluorescent staining images (10 X magnification) were represented, scale bar: 100 µm. (B) Quantification of percentage of NP‐positive cells from eight fields covering a total of 400–600 cells. (C) Viral titer in culture media as determined by plaque assay. Data was expressed as means ± SE. *p < 0.05 (*n* = 3), Student's *t*‐test.

To determine the functional activity of SNHG15 in primary cells, human bronchial tracheal (HBTE) cells were infected with PR8 virus at an MOI of 1 for 24 h and immunostained for viral NP protein. 23.28 ± 0.66% and 45.92 ± 1.03% of NP‐positive cells were observed in VC‐ and SNHG15‐ overexpressed HBTE cells (Figure 5A,B). Also, viral titer levels in media were ~1 log higher in SNHG15‐overexpressed HBTE cells than that in VC cells (Figure 5C).

### 2.5 SNHG15 Competes With miR‐153 to Regulate Influenza Viral Replication

**Figure 6:**
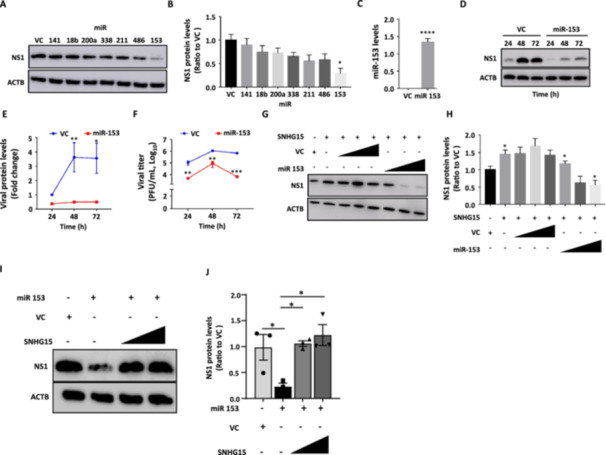
SNHG15 acts through sponging miR‐153. HEK 293 cells were transfected with a miRNA expression vector using Lipofectamine 3000, followed by infection with PR8 at an MOI of 0.01 for 48 h. (A–C). A549 cells were transduced with miR‐153 or vector control (VC) lentivirus at an MOI of 200 for indicated times (D–F). A549 cells were transduced with SNHG15 lentivirus at an MOI of 100 and miR‐153 or VC lentivirus at an increasing MOI of 25, 50, and 100 for 48 h (G, H), or with miR‐153 lentivirus at an MOI of 100 and SNHG15 lentivirus at an MOI of 50 and 100 (F, G). Cells were then infected with 2009 pandemic H1N1 OK/09 and at an MOI of 0.05 for 48 h. miR‐153 level was determined by real‐time PCR (C), and viral NS1 protein were determined by Western blot (A, B, D, E, and G–J). RNA and protein levels were normalized to β‐actin. Viral titer was determined by plaque assay (F). Data was expressed as means ± SE. *p < 0.05, **p < 0.01, ***p < 0.001, ****p < 0.0001 versus VC of corresponding dose (E, H), time (F) or indicated (J) (*n* = 3). One‐way ANOVA followed by Tukey's comparison for B and J, Student's *t*‐test for C, two‐way ANOVA followed by Tukey's comparison for E, F, and H.

One of the mechanisms that lncRNAs function is to compete with endogenous microRNAs. Seven microRNAs namely miR‐141, miR‐18b, miR‐200a, miR‐338, miR‐211, miR‐486, and miR‐153 were previously shown to bind with SNHG15 [45, 46, 47, 48, 49, 50, 51]. To determine whether SNHG15 could regulate influenza viral replication via these microRNAs (miRNAs), we first examined the effects of overexpression of these miRNAs on influenza viral infection. We transfected a miRNA expression vector with a GPF marker in HEK 293 cells and then infected with PR8 virus at an MOI of 0.01 for 48 h. High transfection efficiencies were confirmed for all miRNAs as revealed by GPF images (Supplementary Fig. 1). Among the seven miRNAs, miR‐153 showed a potent antiviral phenotype against PR8 virus strain (Figure 6A,B). The overexpression of miR‐153 was further validated by real‐time PCR (Figure 6C).

We then determined the virus growth kinetics in miR‐153‐overexpressed cells. We overexpressed miR‐153 in A549 cells using a lentiviral vector and infected the cells with pdm OK at an MOI of 0.05 for 24, 48, and 72 h. Overexpression of miR‐153 reduced viral NS1 protein levels at all the time points (Figure 6D,E). Viral titers in media of miR‐153 overexpressed cells were 1.33 ± 0.08, 1.59 ± 0.37, and 2.02 ± 0.18 log lower than these of VC at 24, 48 and 72 hpi (Figure 6F).

To determine whether SNHG15 regulates IAV replication through sponging miR‐153, we overexpressed miR‐153 in SNHG15‐overexpressed A549 cells to see whether miR‐153 can rescue SNHG15 effects on IAV infection. A549 cells were transduced with MOI 100 of VC or SNHG15 along with control or miR‐153 lentivirus at indicated MOI of 25, 50, and 100. After 48 h, cells were infected with pdm OK virus at an MOI of 0.05 for 48 h and viral NS1 protein levels were determined. Compared to VC, SNHG15 overexpressed cells showed an increased viral protein level. miR‐153 overexpression abolished the proviral activity of SNHG15 (Figure 6G,H). Conversely, A549 cells were co‐transduced with VC or miR‐153 lentivirus (MOI = 100) together with SNHG15 lentivirus at the indicated MOIs (50 and 100). Following 48 h of transduction, cells were infected with pdm H1N1 OK/09 virus (MOI = 0.05) for 48 h, and viral NS1 protein levels were analyzed. As expected, miR‐153 overexpression significantly reduced viral NS1 expression compared with VC. Importantly, SNHG15 overexpression effectively reversed the antiviral activity of miR‐153 (Figure 6I,J). Collectively, these reciprocal rescue experiments demonstrate that SNHG15 promotes influenza virus replication by functionally antagonizing miR‐153. Although our reciprocal overexpression rescue experiments support a functional interaction between SNHG15 and miR‐153, additional loss‐of‐function experiments using SNHG15 knockdown combined with miR‐153 overexpression or inhibition will be valuable to further establish that the proviral activity of SNHG15 is mediated through endogenous miR‐153.

### 2.6 SNHG15 Interacts With RABL2A to Regulate Influenza Virus Replication

**Figure 7:**
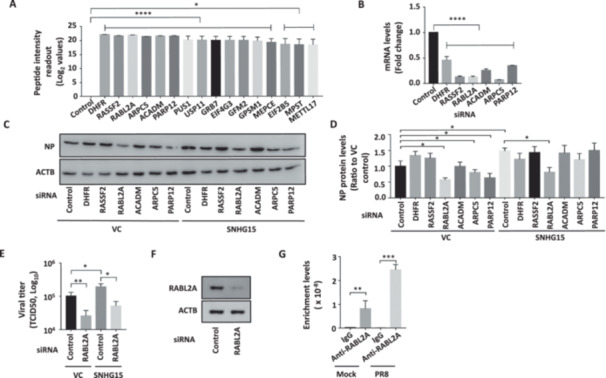
SNHG15 regulates viral infection through RABL2A. SNHG15 interacting protein partners were identified using mass spectrometry analysis (A). A549 cells were transfected with 20 nM of target or control siRNAs using Lipofectamine RNAi max reagent for 48 h, and their mRNA expression levels were determined using real‐time PCR (B). Stable SNHG15 or vector control cells were transfected with 20 nM of target specific siRNAs or control for 48 h and then infected with PR8 at an MOI of 0.01 for 48 h. Viral NP protein levels were determined by western blot and quantified (C, D). Viral titers in media were determined by TCID50 assay (*n* = 4) (E). RABL2A protein levels in stable vector control cells transfected with 20 nM of target specific siRNAs or control for 48 h were determined by western blot (F). SNHG15 levels in the immunoprecipitates with anti‐RABL2A or IgG from mock or PR8‐infected at MOI 1 for 24 h A549 cells were determined using real‐time PCR (G). SNHG15 enrichment levels were calculated using the formula 2^−ct^. Data was expressed as means ± SE. *p < 0.05, **p < 0.01, ***p < 0.001, ****p < 0.0001 (*n* = 6, two independent experiments, each run triplicates for mass spectrometry for A, *n* = 4 for B, D, and *n* = 3 for the rest). One‐way ANOVA, followed by Tukey's comparison for A, Student's *t*‐test for B, Two‐way ANOVA, followed by Tukey's comparison for D, E, and G.

Since SNHG15 is located in the cytoplasm, it may exert its function by interacting with host cytoplasmic proteins. In order to identify the interacting host protein partners of SNHG15, we performed RNA pulldown of A549 cell lysates using biotin‐labeled in vitro transcribed SNHG15. Antisense SNHG15 was used as a control. Two independent experiments were performed, and each pull‐down sample was run for mass spectrometric analysis in triplicates. Mass spectrometry analysis identified a total of 37 proteins that were significantly enriched in SNHG15 pulldown samples with a fold change of > 2. Among those 37 proteins, 16 candidates had a significant readout intensity with SNHG15 but had no or negligible interaction with antisense control (Supplementary Table 3, Figure 7A).

The top six out of the 16 narrowed‐down candidates had ≥ 20 log_2_ peptide readouts in the SNHG15 pulldown samples but zero with antisense control. Thus, we chose the top six hits namely Dihydrofolate reductase (DHFR), Ras association domain‐containing protein 2 (RASSF2), Rab‐like protein 2 A (RABL2A), Medium‐chain specific acyl‐CoA dehydrogenase, mitochondrial (ACADM), Actin‐related protein 2/3 complex subunit 5 (ARPC5), and Poly ADP‐ribose polymerase 12 (PARP12) to further study whether such interactions have any functional consequences. We knocked down each of these interacting partners using siRNA and examined its effect on IAV infection in VC and SNHG15 stable cells. The efficient knockdown was confirmed by real‐time PCR, ranging from 54% to 93% reduction of each gene (Figure 7B). We then infected the knocked‐down cells with PR8 virus at an MOI 0.01 for 48 h and determined viral NP protein levels. Among the interacting partners examined, the knockdown of RABL2A, ACADM, or PARP12 reduced the viral NP levels in the VC control cells. However, only RABL2A knockdown reduced the viral NP protein levels in SNHG15 stable cells (Figure 7C,D). The magnitude of SNHG15‐mediated viral protein induction varied among experiments because of differences in the overexpression systems (Figures 4, 6, and 7D). Nevertheless, all approaches consistently demonstrated that SNHG15 promotes IAV protein expression. Viral titers in the media of RABL2A knockdown cells were also reduced in VC and SNHG15 stable cells (Figure 7E). The reduction of RABL2A protein levels in the knocked‐down cells was confirmed by Western blotting (Figure 7F).

To determine whether influenza virus infection affects the interaction between SNHG15 and RABL2A, we performed an RNA immunoprecipitation (RIP) assay using RABL2A antibodies or IgG control. RIP assay results in mock or PR8 infected‐A549 cells revealed that SNHG15‐RABL2A interaction was increased significantly when compared to the mock condition (Figure 7G).

### 2.7 SNHG15‐RABL2A Interaction Aids Influenza Virus Internalization

**Figure 8:**
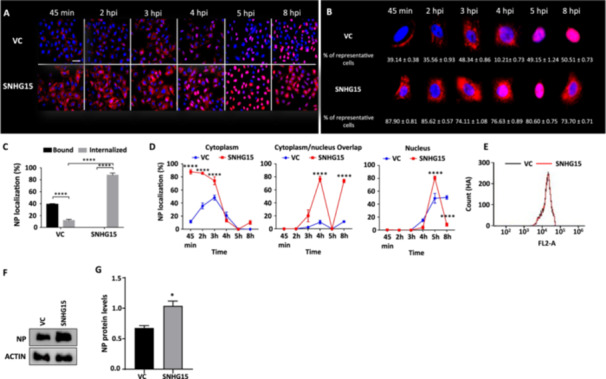
SNHG15 promotes IAV internalization. Stable SNHG15 or vector control cells were infected with PR8 at an MOI of 10 for indicated time points and stained for NP (A–D). NP immunofluorescent staining images for respective time points at 20 X magnification (A) or enlarged single cell NP localization with percentage of representative cells were shown, scale bar: 100 µm (B). A total of 100–150 cells for each condition from six fields for each experiment were counted (A, B). Percentage of represented cells showing NP localization kinetics at indicated time points were quantified (B). Quantification of percentage of the cells with cell surface‐bound, internalized particles at 1 hpi (C), cytoplasmic, cytoplasmic/nuclear overlap or nuclear location of virus particles at indicated times (D). IAV binding efficiency of PR8‐infected VC and SNHG15 stable cells at MOI 10 for 1 h at 4°C in ice as analyzed by flow cytometry for HA (E). A549 cells were transduced with either control vector (VC) or SNHG15‐expressing lentivirus (MOI = 200) for 48 h and subsequently infected with PR8 (H1N1) virus at an MOI of 10 on ice at 4°C for 1 h. Following a temperature shift to 37°C for 30 min to allow viral internalization, cells were washed with ice‐cold PBS–HCl (pH 1.3) to remove surface‐bound virus and lysed. Internalized viral particles were detected by Western blot analysis of NP (F) and quantified by densitometry (G). Data was expressed as means ± SE. *p < 0.05, ***p < 0.001, ****p < 0.0001 vs VC at corresponding time points or as indicated by bars (*n* = 3). Two‐way ANOVA, followed by Tukey's comparison for C and D, and Student's *t*‐test for G.

To determine how SNHG15 promotes influenza virus infection via SNHG15‐RABL2A interaction, we first examined the effects of SNHG15 on the influenza virus life cycle using immunostaining. Stable SNHG15 and VC cells were infected with PR8 virus at an MOI of 10 at various time points for the first cycle of IAV replication and then stained for NP. We quantified > 100 cells for each condition and determined the percentage of NP located at 4 different compartments (Figure 8A,B). (i) cell surface for these with NP present on the cell surface but not entered into the cells, which was typically seen in VC cells at 45 min post infection (Figure 8B), (ii) cytoplasm only, which was typically observed in SNHG15 cells at 45 min‐3 hpi, (iii) cytoplasm and nucleus overlap, which was typically seen in SNHG15 cells at 4 or 8 hpi, and (iv) nucleus only, which was typically seen in VC cells and SNHG16 cells at 5 hpi. The intensities of NP staining were not considered when the cells with a different location were counted. At 45 min post infection, VC cells had 39.14 ± 0.38% of bound virus particles and 11.65 ± 0.63% of internalized virus particles, while 87.90 ± 0.81% of stable SNHG15 cells had NP particles internalized into the cells and none of the cells had the virus particles on cell surface (Figure 8C). The overall intensity and the number of cells with NP staining was higher in SNHG15 cells than VC cells. The results indicate that SNHG15 promotes IAV internalization. In SNHG15 cells, nuclear import was observed at 3 hpi as indicated by the emerge of NP cytoplasmic and nuclear localization and completed at 5 hpi as 81% of the cells had NP nuclear location (Figure 8D). In VC cells, virus particles continued to be internalized up to 3 hpi with nuclear import reaching a maximum at 5 hpi. At 8 hpi, 73.70 ± 0.71% of stable SNHG15 cells exhibited NP cytoplasmic and nuclear location, while NP signals were still inside the nucleus in VC cells, suggesting that SNHG15 may also promote virus nuclear export.

Since the effects of SNHG15 were seen at a very early stage of the IAV life cycle, we determined whether SNHG15 affects the binding of IAV to the cell membrane. Stable SNHG15 and VC cells were infected with PR8 at an MOI of 10 at 4°C for 1 h. The bound virus particles were measured by flow cytometry using anti‐HA. Flow cytometry analysis revealed no difference in the binding of IAV to cell membrane between stable SNHG15 and VC cells (Figure 8E).

In order to quantitatively validate viral internalization, SNHG15‐overexpressing and VC A549 cells were generated by lentiviral transduction and were infected with PR8 virus at an MOI of 10 at 4°C for 1 h to allow viral binding, followed by a temperature shift to 37°C for 30 min to permit internalization. Cells were subsequently washed with ice‐cold PBS–HCl (pH 1.3) to remove surface‐bound, non‐internalized virions. The levels of nucleoprotein (NP) associated with internalized viral particles were then assessed by Western blotting. These results confirmed that SNHG15 enhances influenza virus internalization (Figure 8F,G).

**Figure 9:**
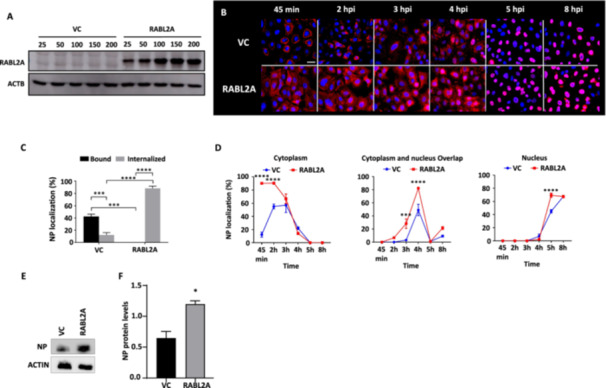
RABL2A promotes IAV internalization. A549 cells were transduced with vector control (VC) or RABL2A lentivirus at indicated MOIs for 48 h and RABL2A protein levels were determined by western blot (A). VC or RABL2A overexpressed A549 cells at an MOI of 100 were infected with PR8 at an MOI of 10 for indicated times (B–D). NP immunofluorescent staining images were represented for respective time points. Scale bar: 100 µm (B). A total of 100–150 cells for each condition from six fields for each experiment were counted (B). Quantification of percentage of the cells with cell surface‐bound, internalized particles at 45 min post infection (C), cytoplasmic, cytoplasmic/nuclear overlap or nuclear location of virus particles at indicated times (D). A549 cells were transduced with control vector (VC) or RABL2A lentivirus (MOI = 200, 48 h) and infected with PR8 (H1N1; MOI = 10) at 4°C for 1 h. After a 30 min shift to 37°C, cells were acid‐washed (PBS–HCl, pH 1.3), lysed, and internalized viral NP was assessed by Western blot (E) and densitometry (F). Data was expressed as means ± SE. *p < 0.05, ***p < 0.001, ****p < 0.0001 vs VC at corresponding time points or as indicated by bars (*n* = 3). Two‐way ANOVA followed by Tukey's comparison for C and D, and Student's *t*‐test for F.

Since RABL2A is a member of RAB GTPases, which are known to regulate the endocytotic pathways during IAV infection [52, 53, 54], we determined the role of RABL2A on IAV life cycle by overexpression of RABL2A. We transduced A549 cells with VC or RABL2A lentivirus at different MOIs for 48 h. Western blot results showed a dose‐dependent increase of RABL2A protein (Figure 9A). We chose a MOI of 100 for further functional studies as the RABL2A expression was saturated at this dose. RABL2A lentivirus‐transduced A549 cells were infected with PR8 virus at a MOI of 10 for indicated time points and stained for NP. Immunostaining results revealed that similar to SNHG15, 90.23 ± 0.47% of RABL2A overexpressed cells had internalized NP particles while only 12.30 ± 0.86% of VC cells had NP internalization and 42.58 ± 0.84% of VC cells had NP particles bound to its cell membrane (Figure 9B,C). The overall percentage and intensity of NP‐positive cells were also higher in RABL2A overexpressed cells. These results indicate that RABL2A promotes IAV internalization into the cells. Unlike SNHG15, RABL2A and VC showed a similar nuclear import and export (Figure 9D), suggesting RABL2A only affects the virus internalization.

Using the internalization assay, we next examined the role of RABL2A in influenza virus entry. RABL2A‐overexpressing A549 cells showed significantly increased levels of internalized viral NP compared with VC cells, as determined by Western blot analysis following acid stripping of surface‐bound virus (Figure 9E,F). These results further support that RABL2A promotes influenza virus internalization.

**Figure 10:**
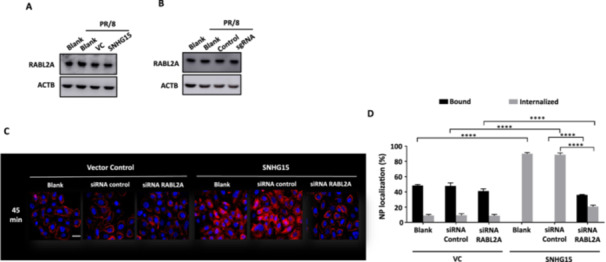
Knockdown of RABL2A blocks SNHG15‐mediated IAV internalization. (A, B) RABL2A protein levels in A549 cells transduced with VC or SNHG15 lentivirus at an MOI of 200 or stable A549‐dCAS9‐KRAB cells transduced with an MOI of 100 of lentivirus carrying sgRNA targeting SNHG15, or its antisense control for 48 h with or without PR8 infection at an MOI of 0.01 for 48 h. RABL2A protein levels were determined by Western blotting. (C, D) Stable SNHG15 or VC cells were transfected with 20 nM of control or RABL2A siRNAs using Lipofectamine RNAi max reagent for 48 h and infected with PR8 at an MOI of 10 for 45 min and stained for NP. Representative images, Scale bar: 100 µm. Quantification of percentage of the cells with cell‐surface bound and internalized particles from a total of 100–150 cells for each condition from six fields for each experiment. Data was expressed as means ± SE. ****p < 0.0001 (*n* = 3). Two‐way ANOVA, followed by Tukey's comparison was used.

Since both SNHG15 and RABL2A showed a correlation in their functional activity on IAV internalization, we further determined the relationship between RABL2A and SNHG15 during IAV infection. We first determined whether SNHG15 affected the RABL2A protein levels. Overexpression or knockdown of SNHG15 did not affect RABL2A protein expression during IAV infection (Figure 10A,B). The results suggest that IAV infection had no effects on RABL2A protein levels.

We next determine whether RABL2A is required for SNHG15‐mediated IAV internalization. We knocked down RABL2A using siRNAs in SNHG15 and VC cells and determined IAV internalization through NP staining at 45 min post infection. In VC cells, no significant differences in cell surface‐bound and internalized virus particles were observed between control and RABL2A knockdown (Figure 10C,D). However, in SNHG15 cells, the cells with internalized virus particles were decreased to 20.80 ± 0.59% in RABL2A knockdown compared to 88.74 ± 0.67% in siRNA control. The cells with cell surface‐bound virus particles were increased to 36.03 ± 0.36% from 0% in siRNA control. These results indicated that RABL2A is required for SNHG15‐mediated IAV internalization. A limitation of this study is that the contribution of endogenous SNHG15 to RABL2A‐mediated viral internalization remains to be fully defined. Future studies using simultaneous endogenous SNHG15 depletion and RABL2A knockdown approaches will be necessary to further clarify the functional relationship between SNHG15 and RABL2A during IAV entry.

## 3 Discussion

The functional significance of new genes is indicated by their evolutionary conservation [55]. In this study, we found that SNHG15 is a conserved lncRNA that was upregulated during influenza virus infection in vitro and in vivo. SNHG15 acts as a proviral lncRNA by competing with the anti‐influenza miRNA, miR‐153‐3p, and enhancing the internalization of viral particles through its association with RABL2A.

SNHG15 is a small nucleolar host gene that encodes SNORA9 within its intron region. According to the human genome assembly (GRCh38/hg38), SNHG15 has five isoforms that share similar sequences and secondary structures but lack protein‐coding potential. SNHG15 was initially identified as a short‐lived lncRNA, and its stability was prolonged in response to external chemical stressors [42]. SNHG15 has been reported to be a critical host factor in promoting cancerous growth in humans, including lung, thyroid, breast, hepatocellular, pancreatic, gastric, and colorectal cancer [56, 57]. While SNHG15 is conserved between humans and mice, its sequence or isoform composition may differ in natural influenza reservoirs, such as birds or swine. Nevertheless, the functional principle of lncRNA‐mediated regulation of host trafficking pathways and miRNA networks may be preserved across species. Future studies examining SNHG15 orthologs or analogous lncRNAs in these hosts, as well as their interaction with conserved host factors like RABL2A, will be important to assess the broader evolutionary relevance of this proviral mechanism.

Our current studies found that SNHG15 isoforms were upregulated by different strains of influenza virus in vitro and in vivo. SNHG15 has been reported to be regulated by MYC transcription factor and was found in high levels in tumors with increased MYC expression [58]. PARP inhibitor Olaparib was also reported to downregulate SNHG15 expression in uveal melanoma cells [59]. A recent study showed that SNHG15 expression was downregulated by EphrinA5‐induced signaling [60]. The mechanisms of how the influenza virus regulates SNHG15 remain to be determined.

Our subcellular localization studies revealed that SNHG15 was predominantly present in the cytoplasm in HBTE cells and A549 cells, and its localization was not changed upon influenza virus infection. This result was consistent with several reports showing that SNHG15 is majorly present in the cytoplasm in various cancerous cell lines, such as HCC, SaoS2, HOS, Pca, and N2a cells and interacts often with microRNAs to exert its functional activities [46, 51, 61, 62, 63, 64, 65, 66]. However, other studies have demonstrated that SNHG15 is located in the nucleus in AsPC‐1 and BxPC‐3 cells and regulates epigenetic modifiers such as H3K27me3 [60, 67]. Thus, the localization of SNHG15 appears to be cell‐type specific.

Our study is the first report showing the functional role of SNHG15 in regulating influenza virus infection. SNHG15 knockdown using CRISPRi decreased influenza virus replication as evidenced by reduced viral RNA, protein, and titer levels. The CRISPRi‐mediated knockdown approach resulted in a reduction of both SNHG15 isoforms and SNORA9 expression levels. However, the observed effects of influenza virus infection by CRISPRi are not due to a decreased SNORA9 because shRNAs targeting the exons of SNHG15 that reduced the expression levels of SNHG15, but not SNOAR9, also decreased influenza virus infection. Furthermore, overexpression of SNHG15 also increased influenza viral replication in A549 and HBTE cells. Our further studies demonstrated that SNHG15 promoted the internalization of influenza viral particles.

Since it is predominantly located in the cytoplasm, SNHG15 may bind with RNAs and proteins to exert its functional activities. Previous studies suggest that SNHG15 potentially binds with several microRNAs [46, 51, 61, 62, 63, 64, 65, 66]. We selected seven microRNAs namely miR‐141, miR‐18b, miR‐200a, miR‐338, miR‐211, miR‐486, and miR‐153 that were validated to target SNHG15 and screened for their activities against influenza virus infection. miR‐153‐3p was our top hit that had a potent antiviral activity against the influenza virus.

miR‐153 has been extensively studied in several cancers, but its role on infectious diseases is not explored extensively [68]. miR‐153 was previously reported to either promote or inhibit tumor growth [68, 69]. miR‐153 is also reported to enhance radiotherapy against pancreatic cancer and enhances CAR T cell immunotherapy against colon cancer [70, 71]. miR‐153 levels were identified to be upregulated in mice with lung sepsis, but down‐regulated during *Helicobacter pylori* and hepatitis B virus infection [72, 73, 74]. The functional role of miR‐153 in these infections is not known.

miR‐153‐3p was previously reported to bind SNHG15 [75, 76, 77]. SNHG15 competes with miR‐153, leading to the increased expression of apoptotic genes such as Bcl‐2, Bax, caspase 3, and cleaved caspase 3 and thus induction of apoptosis in MDA‐MB‐231 cells [51]. SNHG15 also inhibits proliferation, migration, and in vitro tube formation of glioma vascular endothelial cells through the negative regulation of miR‐153 [77]. In an effort to construct a cardiac fibroblast‐related competing endogenous RNA (ceRNA) network, it was revealed that knockdown of SNHG15 increased the expression levels of miR‐153 and further decreased ADAM19 mRNA levels, indicating their potential role in the activation of cardiac fibroblasts [78]. Our rescue experiment revealed that overexpression of miR‐153‐3p abolished the SNHG15‐mediated proviral activity against the influenza virus. The mechanism by which miR‐153‐3p affects IAV replication is not yet revealed. miR‐153‐3p has been shown to negatively regulate CDC42, which has been reported to affect the transport of NA protein to the cell surface [77, 79]. CDC42 has also been shown to interact with YRKL sequence of M1 protein, which is involved in the viral budding process.

Besides microRNAs, we further explored whether SNHG15 binds host proteins. RNA pulldown coupled with proteomics revealed that SNHG15 specifically interacted with 16 host proteins. Based on the peptide readouts, we focused on the top six candidates. We used the siRNA‐mediated knockdown approach to determine the role of these identified binding partners on SNHG15‐mediated proviral activity. Results revealed that RABL2A knockdown significantly hindered the SNHG15's proviral activity.

RABL2A is a member of RAB GTPases, indicating its possible involvement with viral particle internalization. In general, members of RAB GTPases are well‐known regulators of endocytosis pathways [80]. RABL2A structurally resembles RAB proteins due to their presence of the RAB GTPase domain but lacks the c‐terminal prenylation region, which is responsible for membrane insertion of proteins [81]. So far, six RAB‐like proteins have been identified, including RABL2A, RABL2B, RABL3, RABL4, RABL5, and RABL6. A few of these RAB‐like proteins, such as RABL2B, RABL4, and RABL5, have been shown to be involved with the regulation of intraflagellar transport in cilia [82, 83, 84]. RABL2A and RABL2B are highly similar paralogs which are a member of RAB‐GTPase whose function is not well characterized yet [85].

In our study, we showed that RABL2A promoted the internalization of IAV particles into the cells. The knockdown on RABL2A limits the functional activity of SNHG15 on IAV internalization, indicating that SNHG15 acts through RABL2A. RABL2A and its activity on viral entry were first reported in rotavirus infection, which utilizes endocytosis‐mediated internalization, similar to influenza virus [86]. Our previous study has also demonstrated that SNHG15 aided SARS‐CoV‐2 entry via RABL2A [87]. We observed enhanced nuclear import and export in SNHG15 overexpressed cells, and not in RABL2A, thus indicating the involvement of RABL2A only in internalization. RAB5 and RAB7 are two well‐known RAB GTPases that are required for influenza virus entry [52]. RAB11A is another RAB GTPase that was found to be critical for the assembly of genomic segments and budding of IAV particles [88, 89, 90, 91]. While several studies report the role of RAB GTPases in the virus life cycle, the exact molecular mechanism of RABL2A on viral entry and internalization is yet to be understood [92].

Although we have demonstrated a physical interaction between SNHG15 and RABL2A, the functional consequences of this interaction remain unclear. SNHG15 may facilitate viral entry by modulating the availability or activity of RABL2A within endocytic or membrane‐associated compartments, acting as a regulatory lncRNA that fine‐tunes RABL2A‐dependent trafficking pathways. Defining the subcellular compartments in which SNHG15 interfaces with RABL2A will be critical for establishing SNHG15–RABL2A regulatory axis, and future studies employing high‐resolution RNA localization and targeted perturbation of endocytic trafficking pathways will be required to elucidate this mechanism.

Altogether, our studies have highlighted the importance of host non‐coding RNAs in regulating influenza virus infection. We have shown that SNHG15 is a conserved lncRNA that promotes influenza virus replication through competing with anti‐viral miR‐153‐5p and favoring early internalization of viral particles through RABL2A.

## 4 Materials and Methods

### 4.1 Cell Culture

Human alveolar epithelial cell line (A549), Madin‐Darby canine kidney cell line (MDCK), human embryonic kidney cell line (HEK 293), and HEK 293 cells containing SV40 T‐antigen (HEK293T) were purchased from American Type Culture Collection (ATCC, Manassas, VA, USA). Human Bronchial Tracheal Epithelial cells (HBTEC) were purchased from Lifeline Cell Technology (Catalog # FC‐0035, Frederick, MD, USA). A549 cells were cultured in F12K media containing 10% fetal bovine serum (FBS) (Atlanta Biologicals, Flowery Branch, GA, USA) and 0.1% penicillin and streptomycin (PS) solution (Life Technologies Corporation, Carlsbad, CA, USA). HEK 293, HEK293T, and MDCK cells were cultured in DMEM media containing 10% FBS and 0.1% PS. HBTE cells were cultured in BronchiaLife Basal Medium (Catalog # LM‐0007) with BronchiaLife LifeFactors (Catalog #LS‐1047) as per the manufacturer's instructions. dCas9‐ KRAB A549 stable cell line was generated and maintained in F12K containing 10% FBS, 0.1% PS, and 1 μg/mL of puromycin as previously described in [37].

### 4.2 Influenza Virus

IAV/Puerto Rico/8/34 (PR8) was purchased from ATCC (Catalog #VR‐95). IAV/WSN/1933 (WSN), IAV/Oklahoma/3052/09 (pdm OK), and A/Oklahoma/309/2006 (H3N2) were kindly provided by Dr. Gillian Air, University of Oklahoma Health Sciences Center. The PR8‐Gluc virus was kindly provided by Dr. Shitao Li, Tulane School of Medicine. Virus stocks propagation, storage, and titer determination were done as previously mentioned [37].

### 4.3 IAV Infection

A549 or HEK 293 cells were seeded at the indicated cell density in a 12‐well plate and transfected the next day with overexpression or shRNA plasmids or siRNAs or transduced with lentiviral particles at indicated doses for 48 h. Cells were then washed with once with Dulbecco's phosphate‐buffered saline (DPBS) and infected with indicated MOIs of PR8 or pdm OK virus using F12K serum‐free media containing TPCK‐trypsin (0.5 μg/mL) at 37°C and 5% CO_2_ incubator for 1 h. The virus inoculum is then replaced with F12K serum‐free media containing TPCK‐trypsin (0.5 μg/mL). At indicated endpoints, the samples were collected. RNAs were extracted using TRI Reagents (Catalog # TR118, Molecular Research Center, Cincinnati, OH, USA) for gene expression by real‐time PCR. Protein samples were collected using M‐PER Mammalian Protein Extraction Reagent with Protease and Phosphatase Inhibitor Cocktail (1X) (Catalog # 1861281, Thermo Scientific, Rockford, IL, USA). Supernatants were collected and stored at −80°C, and virus titer determination was performed using plaque assay or TCID_50_ assay.

### 4.4 Plaque Assay

MDCK cells were seeded at a density of 5 × 10^5^ cells in a 6‐well plate using 3 mL of DMEM complete media containing 10% FBS and 0.1% PS. The next day cells were inoculated with 800 μL of 10‐fold serially diluted samples ranging from 10^−3^ to 10^−8^ and incubated at 37°C and 5% CO_2_ incubator for 1 h. The inoculum is then replaced with 3 mL of 1:1 dilution of 2 X DMEM serum‐free media and 2% SeaPlaque Agarose (Catalog # 50101, Lonza, Morristown, NJ, USA) overlay mixture containing TPCK‐trypsin (1 μg/mL) and allowed to solidify at room temperature for 30 min. Then the plates are kept upside down at 37°C and 5% CO_2_ incubator for 72 h. Cells are then fixed with 10% neutral buffered formaldehyde, and the plaques are stained with crystal violet (Catalog # HT90132, Sigma‐Aldrich, St. Louis, MO, USA) for 2 min and washed. The wells showing a countable range of 5–50 plaques were counted, and titer (PFU/mL) was calculated using the formula (number of plaques × dilution factor × 1.25).

### 4.5 TCID_50_ Assay

MDCK cells were seeded at a density of 2 × 10^5^ cells in a 96‐well plate using 100 µL of DMEM complete media containing 10% FBS and 0.1% PS. The next day cells were inoculated with 50 μL of 10‐fold serially diluted samples and incubated at 37°C and 5% CO_2_ incubator for 1 h. The inoculum is then replaced with DMEM serum‐free media containing TPCK‐trypsin (1 μg/mL). Cytopathic effects were observed after 5 days post‐infection, and virus titers were calculated according to Reed and Muench method [93].

### 4.6 Isolation of Cytoplasmic and Nuclear RNAs

Cytoplasmic and nuclear RNAs were isolated from A549 cells with or without PR8 infection at an MOI of 1 for 24 h and from HBTE cells using Cytoplasmic and Nuclear RNA Purification Kit (Catalog #21000, Norgen Biotek Corporation, Thorold, ON, Canada). cDNA was prepared using 500 ng RNA, and real‐time PCR was performed. To determine the efficiency of fractionalization, β‐actin (ACTB) and glyceraldehyde 3‐phosphate dehydrogenase (GAPDH) were used as cytoplasmic markers, and U2 small nuclear RNA (U2 snRNA) was used as a nuclear marker. SNHG15 and marker gene levels in individual fractions were calculated using the formula 2^−ct^ and then multiplied by a dilution factor (total ng of RNA in each fraction/500). The distribution percentage of each gene was calculated as a target gene amount in an individual fraction divided by the sum of target gene amounts in the cytoplasm and nuclei and multiplied by 100.

### 4.7 Construction of Vectors

#### 4.7.1 Overexpression Vectors

Using Kapa Hotstart HiFi PCR kit (Roche, Basel, Switzerland) SNHG15 isoform 1 was PCR‐amplified from human genomic DNAs from A549 cells and RABL2A from RABL2A pLX304 plasmid (Catalog No. HsCD00440413, DNASU Plasmid Repository, Tempe, AZ, USA) with the primers listed in Supplementary Table 4. The thermal temperatures were: 95°C for 3 min, followed by 35 cycles of 98°C for 20 s, 60°C for 15 s, 72°C for 60 s, and a final extension of 72°C for 1 min. SNHG15 PCR products were inserted into a modified lentiviral vector pLVX (Clontech, Mountain View, CA, USA) downstream of its green fluorescent protein (GFP) at XhoI and EcoRI and at NotI and XhoI for RABL2A.

#### 4.7.2 miRNA Overexpression Vectors

Mature miRNA plus flanking sequences were amplified from human genomic DNAs using the primers listed in Supplementary Table 4 as previously described in [94]. The PCR products were inserted into a modified lentiviral vector pLVX (Clontech) downstream of its green fluorescent protein (GFP) at XhoI and EcoRI.

#### 4.7.3 CRISPR Interference Vectors

Using CHOPCHOP software (https://chopchop.cbu.uib.no/), a single guide RNA (sgRNA) specifically targeting the promoter region of SNHG15 was designed. SNHG15 sgRNA 5′‐CACCGAGGAATGGTCAGGCAACACG‐3′ was cloned into the lentiGuide‐Puro vector (Addgene, Cat# 52963, Watertown, MA, USA) for expressing hU6‐driven sgRNA using BsmBI sites. 5′‐GGTGGTAGAATAACGTATTAC‐3′ was used as the sgRNA control sequence.

#### 4.7.4 shRNA Vectors

Using the BLOCK‐iT RNAi Designer, shRNA sequences targeting the exon 4 of SNHG15 isoforms were designed. Two shRNAs sequences were designed and inserted into the hCMV promoter‐driven lentiviral miRZip vector (System Biosciences, Palo Alto, CA, USA) at the downstream of its GFP at BamH1 and EcoR1. The shRNA sequences for SNHG15 are listed in Supplementary Table 4. All inserts were confirmed by DNA sequencing.

### 4.8 Lentivirus Production and Titer Determination

Lentivirus particles were produced in HEK293T cells as previously described. Titer determination was done as previously mentioned [37, 94]. The number of infectious units (IU) for overexpression lentivirus particles with EGFP was calculated based on the number of EGFP‐positive cells, and for CRISPR interference lentivirus particles without EGFP using a colony formation assay.

### 4.9 Cell Viability Assay

A549 cells were seeded at a density of 2.5 × 10^4^ cells per well in a 96‐well plate. The next day, cells were transfected with shRNAs using Lipofectamine 3000 reagent (Catalog # L3000015, Thermo Fischer, Waltham, MA, USA) as per the manufacturer's instructions. After 48 h, 20 µL of cell titer blue reagent (Catalog # G8080, Promega, Madison, WI, USA) was added to the cells, and OD values were measured at 570 nm.

### 4.10 siRNA Transfection

siGENOME Human RABL2A siRNA SMARTPool (Catalog # M‐013620‐00‐0010) and siGENOME non‐targeting siRNA Control Pool #2 (Catalog # D‐001206‐14‐20) were purchased from Dharmacon (Lafayette, CO, USA). A549 cells (0.1 × 10^5^) were seeded in each well of 12‐well plates. 24 h post‐seeding, 20 nM of RABL2A or control siRNA was transfected using Lipofectamine RNAi Max transfection reagent (Catalog # 13778075, Thermo Fischer) as per the manufacturer's instructions. 48 h post‐transfection, cells were collected for RNA and protein extraction.

### 4.11 Real‐Time PCR

RNA samples were collected in TRI reagent and RNA isolation was performed as previously described in [37]. One µg of the total RNA was reverse‐transcribed into cDNA using the MMLV enzyme (Thermo Fischer). Primer3 software was used to design primers for target genes. Primer sequences were listed in Supplementary Table 4. Real‐time PCR was performed using iTaq Universal SYBR Green Supermix (Catalog # 1725124, Bio‐Rad, Hercules, CA, USA) on QuantStudio 6 Real‐Time PCR System (Thermo Fischer). The thermal temperatures were: 95°C for 10 min, followed by 40 cycles of 95°C for 15 s, 60°C for 30 s, and 65°C for 30 s. ACTB was used as the endogenous reference gene. Relative gene expression levels were calculated by the comparative ΔCt method using the equation 2^−ΔCt^.

### 4.12 Western Blot

Samples were collected in T‐PER Tissue Protein Extraction Reagent (Catalog # 78510, Thermo Fischer) with 1X Halt Protease and Phosphatase Inhibitor Cocktail. Protein concentrations were measured using Bio‐Rad Protein Assay (Catalog No 5000006, Bio‐Rad, Hercules, CA, USA). Ten µg of protein samples were run in 10% SDS‐PAGE gel and transferred to nitrocellulose membranes using Trans‐Blot Turbo Transfer System (Bio‐Rad,). After transfer, the membranes were washed three times with 1X Tris‐buffered saline (pH 7.5) and 0.05% Tween 20 (TBST Buffer), blocked with 5% skim milk for 1 h, and incubated overnight with primary antibodies for at 4°C and washed thrice with TBST Buffer. Then secondary antibodies were added and incubated for 1 h at room temperature and washed thrice with 1 X TBST Buffer. The following primary and secondary antibodies were used: mouse anti‐NP, 1:40 dilution (Catalog # HB‐65, ATCC), mouse anti‐NS1, 1:1000 dilution (Catalog #, SC‐130568, Santa‐Cruz Biotechnology, Dallas, TX, USA), mouse anti‐β‐actin, 1:3000 dilution (Catalog # MA5‐15739, Thermo Scientific), rabbit anti‐RABL2A, 1:1000 dilution (Catalog # 17816‐1‐AP, Proteintech, Rosemont, IL, USA), goat anti‐rabbit HRP‐conjugated second antibody, 1:1000 dilution (Catalog # 111‐035‐003, Jackson Immuno Research Laboratories, West Grove, PA, USA), and goat anti‐mouse HRP‐conjugated secondary antibodies 1:2000 (Catalog #115‐0.5‐003, Jackson Immuno Research Laboratories). Super Signal West Pico Chemiluminescent Substrate (Thermo Fischer) was used to visualize target proteins, and images were taken with Amersham Imager 600 (GE Healthcare System, Pittsburgh, PA, USA).

### 4.13 Luciferase Reporter Assay

HEK293 cells were seeded at a density of 2 × 10^4^ cells/well in a type I collagen‐coated 96‐well plate and transfected with an influenza virus luciferase reporter vector, pHH21‐NP‐3′‐UTR‐LUC‐NP‐5′‐UTR (20 ng), SNHG15 shRNA plasmids (100 ng), and a pRL‐TK vector (5 ng) using Lipofectamine 3000 reagent. The next day, cells were infected with the influenza virus, PR8 (MOI 0.1) or pdm OK (MOI 0.5) for 24 h. Then, cells were lysed using 1x passive lysis buffer, and luciferase activities were measured using a dual luciferase assay kit (Catalog # E1910, Promega) as per the manufacturer's instructions. Results were expressed as the ratio of firefly to *Renilla* luciferase activities.

### 4.14 Gaussia Luciferase Assay

A549 cells were seeded at a density of 1 × 10^5^ cells in a 24‐well plate and transduced with VC and SNHG15 lentivirus particles at an MOI of 200. 48 h post‐transduction, cells were infected with PR8 Gluc virus at an MOI of 1 for 24 h. Cell lysates were collected using 1X cell lysis buffer. Luciferase activities were measured using Pierce Gaussia Luciferase Flash Assay Kit (Catalog # 16158, Thermo Scientific) as per the manufacturer's instructions. Results were expressed as relative light units (RLU).

### 4.15 Immunofluorescence Staining

For HBTE staining, cells were seeded at a density of 3 × 10^5^ cells in a 24‐well plate and transduced with VC and SNHG15 lentivirus particles at an MOI of 200. 48 h post‐transduction, the cells were infected with PR8 at an MOI of 1 for 24 h using Broncho Life Basal media. Cells were fixed with 4% paraformaldehyde for 15 min at room temperature.

For virus internalization and life cycle studies, 2.5 × 10^4^ cells stable SNHG15 and vector control cells with or without RABL2A or control siRNA were seeded in a 24 well‐plate. 12 h post‐seeding, cells were infected with PR8 virus at an MOI of 10 for indicated times and fixed with 4% paraformaldehyde for 15 min at 37°C. Cells were washed once with DPBS, and permeabilization was done using 0.1% Triton X‐100 for 20 min at room temperature. Cells were washed again once with DPBS and blocked with 10% normal goat serum for 1 h at 37°C. Primary antibody‐containing 1:20 dilution of monoclonal mouse anti‐NP (Catalog # HB‐65, ATCC) in 10% of normal goat serum was added and incubated for 1 h at 37°C. Then cells were washed twice with DPBS and then incubated with Alexa fluor 546‐conjugated polyclonal goat anti‐mouse IgG antibodies (1:300, Life technologies, Carlsbad, CA, USA) for 1 h at 37°C. Without removing the secondary antibody, Hoechst 3342 at a concentration of 2 µg/mL (Molecular probes, Waltham, MA, USA) was added and incubated at 37°C for 10 min. Cells were then washed thrice with DPBS, and images were taken in a fluorescence microscope using Meta Imaging Series 7.7. At least six fields covering 100–150 cells for each condition in an individual experiment were counted and analyzed. NP staining was classified into the following categories based on their location: cell surface‐bound, cytoplasm, nucleus, and cytoplasm/nucleus overlap. NP staining present on the cell surface but not internalized into the cells are considered to be cell surface‐bound particles, whereas internalized particles that are present only in the cytoplasm or nucleus are considered to be cytoplasmic or nuclear localization. Cells showing the presence of NP staining both in cytoplasm and nucleus irrespective of the intensity are considered to be cytoplasm/nucleus overlap.

### 4.16 RNA Pulldown and Mass Spectrometry

MEGAscript T7 Transcription Kit (Catalog # AM1330, Thermo Fischer) was used to generate in vitro transcribed SNHG15 isoform 1 and its antisense control with the primers listed in Supplementary Table 4 according to the manufacturer's instructions. Fifty pmol of SNHG15 isoform 1 and antisense control were biotin‐labeled using the Pierce RNA 3' Desthiobiotinylation Kit (Cat # 20163, Thermo Scientific) according to the manufacturer's instructions. Fifty pmol of labeled RNAs were incubated with 200 µg of total proteins from A549 whole cell lysates, and RNA pulldown was performed using Pierce Magnetic RNA‐Protein Pull‐Down Kit (Cat # 20164, Thermo Scientific) according to the manufacturer's instructions. Eluted samples were analyzed using Orbitrap Mass Spectrometry at the Protein Core Facility, Oklahoma State University. Two independent RNA pull‐downs were performed, and 3 replicates for each sample were run on Orbitrap Mass Spectrometry. Peptide intensities were measured.

### 4.17 RNA Immunoprecipitation Assay

Approximately 2 × 10^7^ of A549 cells were harvested, washed once with DPBS, and lysed using 1 mL of RIP buffer containing 1x protease and phosphatase inhibitor cocktail and 100 U/mL of RiboLock RNase inhibitor (Cat # EO0382, Thermo Scientific). To the cell lysate, 1 µL g of rabbit IgG control (Catalog # 2729S, Cell Signaling Technology, Danvers, MA, USA) and 40 µL of prewashed Protein A/G PLUS‐Agarose beads (Catalog # sc‐2003, Santa Cruz Biotechnology, Dallas, TX, USA) were added and incubated at 4°C for 1 h at 30 rpm using Stuart SB3 rotator (TE Equipment, Long Branch, NJ, USA) in order to preclear the lysate. After 1 h of incubation, beads were pelleted by centrifugation at 1000 × g for 5 min at 4°C. For input control, 50 µL of supernatant was taken and 1 mL of TRI reagent was added. 5 µg of Rabbit IgG control and RABL2A antibodies (Catalog # 17816‐1‐AP, Proteintech, Rosemont, IL, USA) were incubated with 350 µL of precleared lysate 4°C at 30 rpm in a rotator overnight. Beads were pelleted using centrifugation at 1000 × g for 5 min at 4°C. Pellets were washed thrice with RIP buffer and once with DPBS. Supernatants were discarded, and 1 mL of TRI reagent was added to the beads. RNAs were isolated from input and immunoprecipitated samples at the same time. Real‐time PCR was run with cDNA prepared from 500 ng of input and total RNA from immunoprecipitated samples. SNHG15 levels were calculated using the formula 2^−ct^.

### 4.18 Animal Samples

Eight‐week‐old C57BL/6 J mice were infected with 250 PFU of the mouse‐adapted strain PR8. Lungs were collected 3 and 7 days post‐infection as described in [94]. RNA samples were obtained from snap‐frozen lungs using TRI reagent. One µg of RNA samples was used for cDNA preparation. The animal procedures were approved by the Institutional Animal Care and Use Committee (IACUC) at Oklahoma State University.

## Author Contributions

S.P. designed and performed experiments, analyzed data, and wrote the manuscript; C.H. performed RNA sequencing analysis and helped in designing and construction of vectors; Y.L. provided special reagents and cell lines; D.X created shRNAs and lentiviruses; G.B., K.V., P.J., K.S., and E.V. assisted with some of the experiments. L.L. conceived the studies, designed experiments, interpreted data and revised the manuscript.

## Conflicts of Interest

The authors declare no conflicts of interest.

## Supporting information


Supporting File

**Table S1:** List of conserved lncRNAs dysregulated during IAV infection

**Table S2:** List of conserved lncRNAs upregulated in mice during influenza infection

**Table S3:** List of SNHG15 interacting partners

**Table S4:** Primers used in this study

## Data Availability

The data that support the findings of this study are available from the corresponding author upon reasonable request.
