# Supplementary material for: SNHG15 Positively Regulates Influenza Virus Infection Through Its Association With miR‐153 and RABL2A
Source: J Med Virol. 2026 Jul 23;98(8):e71084. doi: 10.1002/jmv.71084 (PMC13396821; doi:10.1002/jmv.71084)
Supplement: Supplementary file 1 — Supporting File [file JMV-98-e71084-s004.pdf]

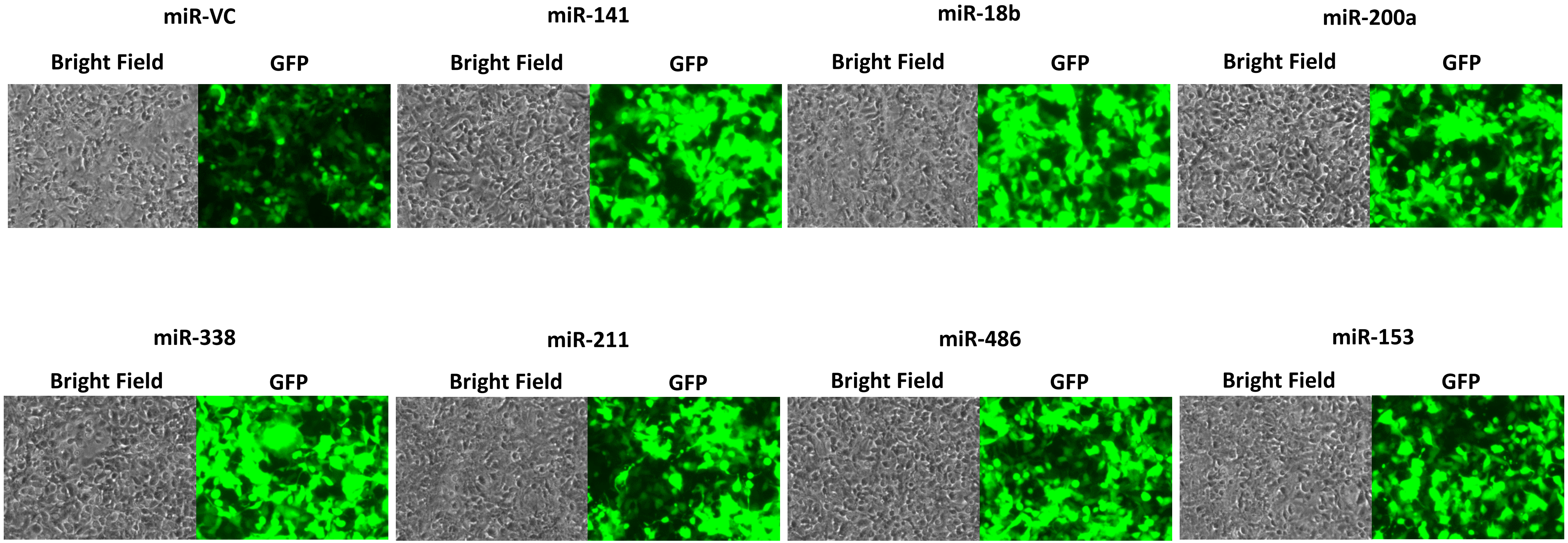

Supplementary figure 1. Transfection efficiency of miRNA candidates. HEK293 cells were transfected with 1.25 µg of selected miRNA candidates and vector control plasmids using lipofectamine 3000for 24 h and followed by PR/8 virus infection at MOI 0.01 for 48 h. Images were taken 72 h post transfection.
